# Identification and Biological Characterization of *Leishmania (Viannia) guyanensis* Isolated from a Patient with Tegumentary Leishmaniasis in Goiás, a Nonendemic Area for This Species in Brazil

**DOI:** 10.1155/2015/350764

**Published:** 2015-10-25

**Authors:** Alause da Silva Pires, Arissa Felipe Borges, Adriano Cappellazzo Coelho, Miriam Leandro Dorta, Ruy de Souza Lino Junior, Ledice Inacia de Araújo Pereira, Sebastião Alves Pinto, Milton Adriano Pelli de Oliveira, Grazzielle Guimarães de Matos, Ises A. Abrahamsohn, Silvia Reni B. Uliana, Glória Maria Collet de Araújo Lima, Fátima Ribeiro-Dias

**Affiliations:** ^1^Instituto de Patologia Tropical e Saúde Pública, Universidade Federal de Goiás, Rua 235 S/N, Setor Universitário, 74605-050 Goiânia, GO, Brazil; ^2^Instituto de Ciências Biomédicas, Universidade de São Paulo, SP, Brazil; ^3^Instituto Goiano de Oncologia e Hematologia e Faculdade de Medicina, Universidade Federal de Goiás, GO, Brazil; ^4^Universidade de São Paulo, SP, Brazil

## Abstract

The aim of this study was to characterize clinical field isolates of *Leishmania* spp. obtained from patients with American Tegumentary Leishmaniasis (ATL) who live in Goiás state, Brazil. The presumed areas of infection were in Goiás, Tocantins, and Pará states. Three isolates of parasites were identified as *L. (Viannia) braziliensis* and one as *L. (V.) guyanensis*. The *in vitro* growth profiles were found to be similar for all parasites. Nevertheless, in C57BL/6 mice, *L. (V.) guyanensis* infection was better controlled than *L. (V.) braziliensis*. Yet in C57BL/6 mice deficient in interferon gamma, *L. (V.) guyanensis* lesions developed faster than those caused by *L. (V.) braziliensis* isolates. In BALB/c mice, the development of lesions was similar for isolates from both species; however, on the 11th week of infection, amastigotes could not be observed in macrophages from *L. (V.) guyanensis*-infected mice. Thus, *L. (V.) guyanensis* can be circulating in Goiás, a state where autochthonous cases of this species had not yet been reported. Considering the difficulties to differentiate *L. (V.) guyanensis* from *L. (V.) braziliensis* at the molecular, morphological, and clinical (human and murine models) levels, the presence of *L. (V.) guyanensis* infections is possibly underestimated in several regions of Brazil.

## 1. Introduction

Leishmaniases are protozoan diseases caused by more than 20* Leishmania* species, which are transmitted by about 30 species of phlebotomine sand flies. Human infections cause three strikingly different clinical presentations and numerous clinical varieties ranging from asymptomatic to disfiguring forms of tegumentary and potentially fatal visceral leishmaniasis. American Tegumentary Leishmaniasis (ATL) presents a spectrum of clinical manifestations characterized by cutaneous (CL), mucosal (ML), disseminated (DL), and diffuse cutaneous leishmaniasis (DCL) [1, 2]. Brazil together with other nine countries accounts for 70–75% of estimated CL cases in the world [3]. A report of the Brazilian Secretary's Office of Surveillance in Health showed a geographic expansion of ATL during the 1980s from the Northern towards the Southern region, and, in 2003, all Brazilian states registered autochthonous cases [4]. In 2013, the distribution per Brazilian region was reported as follows: Northern region with 8,407 new cases (49.5 cases per 100.000 population); Central Western region with the second highest prevalence, 2,922 new cases (19.5 cases per 100.000 population), Northeastern region with 5,355 new cases (9.6 cases per 100.000 population), Southeastern region with 1,150 new cases (1.4 cases per 100.000 population), and Southern region with 296 new cases (1.0 case per 100.000 population) [5, 6].

Three main* Leishmania* species are responsible for ATL in Brazil:* L. (Viannia) braziliensis*,* L. (Leishmania) amazonensis*, and* L. (Viannia) guyanensis*. Besides,* L. (Viannia) lainsoni*,* L. (Viannia) naiffi*, and* L. (Viannia) shawi* have also been identified as new agents of ATL in the Northern region. The species* L. (V.) braziliensis* presents wider geographic distribution than the other species in Brazil (it is reported in all Brazilian states) whereas* L. (V.) guyanensis* is believed to be restricted to the Northern region [7, 8]. The distribution of* Leishmania* species depends on the vectors, animal reservoirs, and hosts as well as the ecology of the endemic areas. As* L. (V.) braziliensis* is widely distributed in South America, this species may be transmitted by several different sand flies species and different animal species can be the reservoirs in distinct ecologic and geographical areas, which increases the molecular diversity of the parasites [9, 10]. In Brazil,* L. (V.) braziliensis* is commonly transmitted by* Lutzomyia whitmani* (Northeastern, Central Western, and Southeastern regions),* L. wellcomei* (Northern region),* L. intermedia* (Southeastern region), and* L. neivai* (Southern region). Besides,* L. umbratilis* has also been suggested as vector for* L. (V.) braziliensis* in Mato Grosso state (Central Western region). The latter species is the main vector for* L. (V.) guyanensis*, which is also transmitted by* L. anduzei* and* L. whitmani* [1, 9, 11, 12]. In Goiás state (Central Western region),* L. intermedia* and* L. whitmani* have been associated with ATL [13, 14]. Mammal reservoirs of* L. (V.) braziliensis* can be found among numerous species of forest animals, especially rodents, whereas* L. (V.) guyanensis* is more frequent in sloths, anteaters, and opossums [8].

*L. (V.) braziliensis* and* L. (V.) guyanensis* are associated with the same clinical manifestations of ATL as localized cutaneous, disseminated, and mucosal leishmaniasis can be ascribed to both species [15–18]. Therefore, determining the* Leishmania* species causing disease in a patient cannot rely on clinical criteria and parasite identification is essential to prescribe the best species-specific therapeutic regimen [15, 17]. Furthermore, genetic heterogeneity and clonal diversity, which leads to variability in parasite virulence, are also common among* L. (Viannia)* spp. parasites [10, 19, 20].

In the present study, we characterized four* Leishmania* spp. isolates obtained from patients with ATL assisted at the Tropical Disease Hospital of Goiânia, Goiás, Brazil, a reference center for leishmaniasis diagnosis and treatment in Goiás state (Central Western region). The leishmaniasis cases from Northern and Central Western Brazil are referred to this hospital. The patients in our study were probably infected in Goiás (Central Western region), Tocantins, or Pará (Northern region) states. A comprehensive knowledge of the species and the characteristics of the parasites are very important for controlling the disease, mainly when patients migrate to other regions/states with different ecosystems and increase the threat of new* Leishmania* foci.

## 2. Materials and Methods

### 2.1. Mice

Female C57BL/6 (wild-type [WT]) or C57BL/6 IFN-gamma knockout (IFN*γ* KO C57BL/6) and BALB/c mice, six to eight weeks old, were obtained from the breeding animal facilities of the IPTSP/Federal University of Goiás, Goiânia, Brazil. All the animal handling and procedures were approved by the Ethical Committee from Clinical Hospital of the Federal University of Goiás on the ethical handling of research animals.

### 2.2. Patients

Four patients were assisted at the Tropical Disease Hospital (Anuar Auad, Goiânia, Goiás, Brazil) with the diagnostic hypothesis of leishmaniasis. All of them live in Goiás, but the presumed areas of infection were Goiás, Tocantins, and Pará/Maranhão border. Diagnosis of ATL was confirmed by epidemiological, clinical, and laboratory analyses. The patients' data are presented in Table 1. The protocols on this investigation relative to human patients and animals were approved by the Ethical Committee from Hospital das Clínicas, Universidade Federal de Goiás, and all patients signed an informed consent form.

### 2.3. Parasite Isolation and Cultures

A fragment of the cutaneous lesion biopsy was macerated and inoculated in mice footpads (IFN*γ* KO C57BL/6) or directly cultured at 26°C in Grace's Insect Cell Culture Medium (Gibco-BRL Life Technologies, Grand Island, NY, USA) containing 20% heat-inactivated fetal bovine serum (Cripion, Andradina, SP, Brazil), 2 mM glutamine, penicillin (100 U/mL), and streptomycin (100 *μ*g/mL) (supplements and antibiotics were purchased from Sigma Chemical Co., St. Louis, MO, USA). After 1-2 months draining lymph nodes from the animals were processed as described [21] and cultured in complete Grace's medium. After being expanded in culture, the parasite isolates were cryopreserved in liquid nitrogen. The parasite isolates were thawed and expanded once in complete Grace's medium before use in all experiments. All these procedures were previously described [21]. The parasite isolates were coded as WWS5 (MHOM/BR/2005/WSS5), UAF5 (MHOM/BR/2005/UAF5), HPV6 (MHOM/BR/2006/HPV6), and PLR6 (MHOM/BR/2006/PLR6). The isolates WWS5, UAF5, and HPV6 were previously identified as* L. (V.) braziliensis* [22, 23].

### 2.4. Molecular Characterization: Polymerase Chain Reactions (PCR)

The identification of the isolates was based on three strategies: (1) small subunit ribosomal RNA (SSU rDNA) was sequenced as previously described, using primers S12/S4 [24]. Positive control reactions were performed using a reference genomic DNA purified from axenic cultures of* L. (L.) amazonensis* MHOM/BR/1973/M2269,* L. (V.) braziliensis* MHOM/BR/1975/M2903,* L. (L.) chagasi* MHOM/BR/1972/LD,* L. (V.) guyanensis* MHOM/BR/75/M4147, or* L. (V.) shawi* MCEB/BR/84/M8408, while in negative controls no genomic DNA was added. The amplified product was analyzed in a 2% agarose gel electrophoresis stained with ethidium bromide. The nucleotide sequence of the 520 bp fragment was obtained directly by automatic sequencing using an ABI Big-Dye kit as described [23]. (2) Sets of primers were used in PCR assays to discriminate* L. (V.) braziliensis* (G6PD-ISVC and G6PD-ISVB) from other organisms of the* Viannia* subgenus (G6PD-ISVG and G6PD-LVF) as described before [25]. PCR reactions were prepared in 50 *μ*L final volume containing 50 mM KCl, 1.5 mM MgCl_2_, 10 mM Tris-HCl (pH 8.3), 0.2 mM of each deoxyribonucleotide, 15 pmol of each primer, 2.5 U of Taq DNA polymerase (Gibco), and 100 ng of template DNA. (3) The ribosomal internal transcribed spacer (ITS) was amplified using primers IR1 and IR2 [26]. The approximately 1 kb PCR amplified product was digested with* Hae* III as described [27] and analysed by gel electrophoresis. The amplified product was also purified from agarose gels using the QIAquick PCR purification kit (Qiagen, Valencia, USA) and cloned in pGEM-T easy (Promega Corporation, Madison, WI, USA). The nucleotide sequence of three independent positive clones, confirmed previously by restriction analysis, was determined as described above using pUC/M13, IR1, IR2, 5.8F (5′ GCAGTAAAGTGCGATAAGTGG 3′), and 5.8R (5′ GGAAGCCAAGTCATCCATC 3′) primers. Nucleotide sequence analyses were performed using Lasergene Software (DNASTAR) and Clone Manager 9.0 Software. Phylogenetic analysis was performed using RAxML [28].

### 2.5. *In Vitro* Growth of* Leishmania* Isolates

The parasite isolates extracted from draining lymph nodes of infected IFN*γ* KO C57BL/6 mice were cultured at an initial concentration of 5 × 10^5^/mL in 24-well culture plates (TPP, Techno Plastic Products, Trasadingen, Swizerland) in complete Grace's medium at 26°C. Samples of parasites were counted daily for 13 days in a hemocytometer after dilution in 2% formaldehyde solution in PBS, under light microscopy.

### 2.6. Infection of Mice

Groups of four mice were injected subcutaneously (s.c.) with 5 × 10^6^ live promastigotes (50 *μ*L) in stationary phase of growth into the left hind footpad. Lesion development was followed by measuring the thickness of the infected paw with a dial caliper at weekly intervals and expressed by the arithmetic mean and standard error mean (SEM) of the net thickness increase (infected minus control contralateral paw thickness). Following ethical procedures, when the paw lesion reached 5 mm in thickness or presented ulceration, the mice were euthanized.

### 2.7. Tissue Processing for Optical Microscopy

To analyze the local inflammatory reaction and presence or absence of parasites, footpads were removed postmortem on the 5th or 6th (IFN*γ* KO C57BL/6) or 11th (C57BL/6 WT, BALB/c) week after infection, excised, and prefixed with 10% formalin, followed by fixation in Bouin solution (picric acid 75%, glacial acetic acid 5%, and formaldehyde 10%) prior to paraffin embedding. Five *μ*m sections from the material were stained with hematoxylin and eosin (H&E) and examined under light microscopy.

### 2.8. Statistical Analysis

Data are presented as mean ± standard error of the mean (SEM). Two way ANOVA/Bonferroni was used to compare the data, and the differences were considered significant when *P* < 0.05.

## 3. Results

### 3.1. Patient Profiles

The age of patients varied from 19 to 46 years and they had one to three cutaneous lesions located in the limbs that appeared from two to eight months before the diagnosis. All patients were diagnosed with ATL, presenting the cutaneous localized clinical form (LCL) according to clinical and laboratory analyses. In all patients, the lesions were ulcerated. Patients' data are presented in Table 1.

### 3.2. Molecular Characterization of* Leishmania* Isolates

SSU rDNA amplification was performed on four clinical field isolates and controls, using primers S12/S4 and the PCR products were analyzed by automatic sequencing. The nucleotide sequence of the four isolates identified HPV6, UAF5, WSS5, and PLR6 as species of the* Viannia* subgenus (data not shown). All samples were also analyzed by PCR of the* G6PD* gene with primers specific for* L. (V.) braziliensis* or “non-*braziliensis*”* Viannia* species. This analysis confirmed the identity of HPV6, UAF5, and WSS5 as* L. (V.) braziliensis* and of PLR6 as a “non-*braziliensis*” isolate (data not shown).

**Figure 1:**
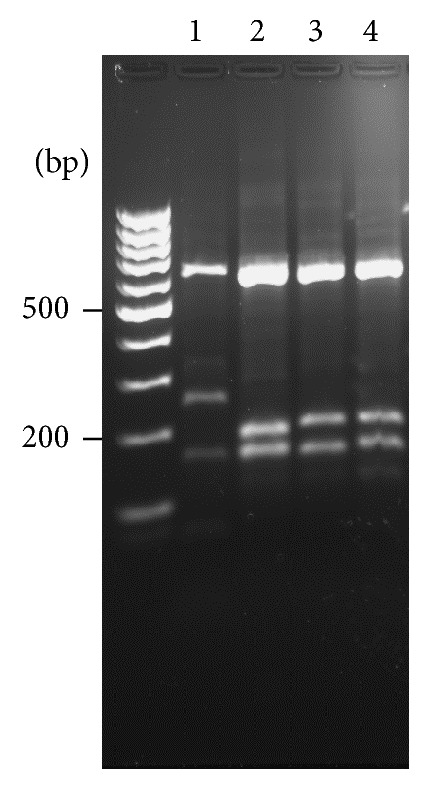
Identification of* Leishmania* PLR6 isolate by ITS1 and 2 amplifications from genomic DNA. The PCR amplified products of approximately 1 kb were digested with* Hae* III and restriction fragment analysis was evaluated in an ethidium bromide stained 2% agarose gel. 1:* L. (L.) amazonensis*, 2:* L. (V.) braziliensis*, 3:* L. (V.) guyanensis*, and 4: PLR6 isolate.

The identification of the PLR6 isolate was based on the analysis of ribosomal ITSs 1 and 2. Approximately 1 kb fragment was amplified and digested with* Hae* III. The analysis of restriction fragment polymorphisms indicated that PLR6 displayed a pattern compatible with* L. (V.) guyanensis* (Figure 1). This was confirmed by nucleotide sequencing of the 1 kb fragment encompassing ITS1, 5.8S rDNA, and ITS2. The sequence obtained (Genbank number AJ000299.1) showed 99% identity with* L. (V.) guyanensis* (MHOM/BR/75/M4147).

### 3.3. Behavior of the Isolates in* In Vitro* Culture

**Figure 2:**
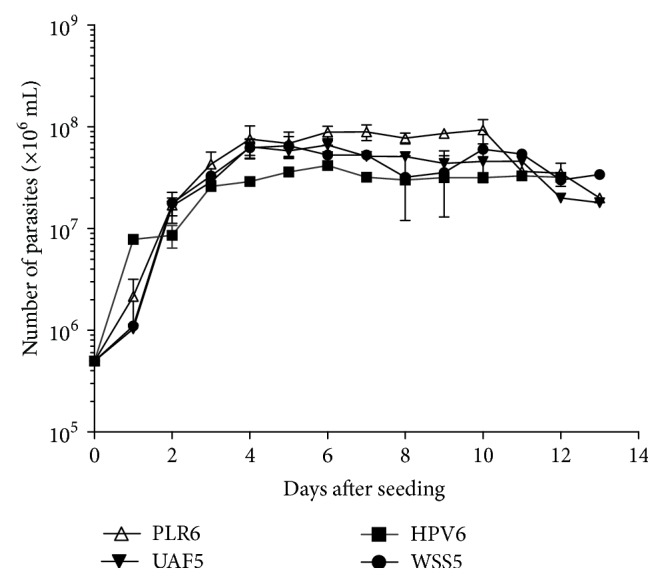
*In vitro* growth curves of* Leishmania (V.) braziliensis* (HPV6, UAF5, and WSS5) and* Leishmania (V.) guyanensis* (PLR6) in complete Grace's medium. Parasites were seeded in 5 × 10^6^/mL and cultured during 13 days, at 26°C in BOD. The data represent mean ± SEM of two-to-three independent experiments performed in triplicate.

Replication rates of the four isolates were similar in complete Grace's medium at 26°C during 13 days. The growth curves exhibited typical logarithmic and stationary phases. The parasites formed large clumps at the stationary phase (data not shown). The maximum number of parasites occurred within 4 to 6 days, ranging from around 5 × 10^7^ to 1 × 10^8^/mL (Figure 2). After 10 days of culture, parasites of all isolates began to die.

### 3.4. Course of Infection in Mice

**Figure 3:**
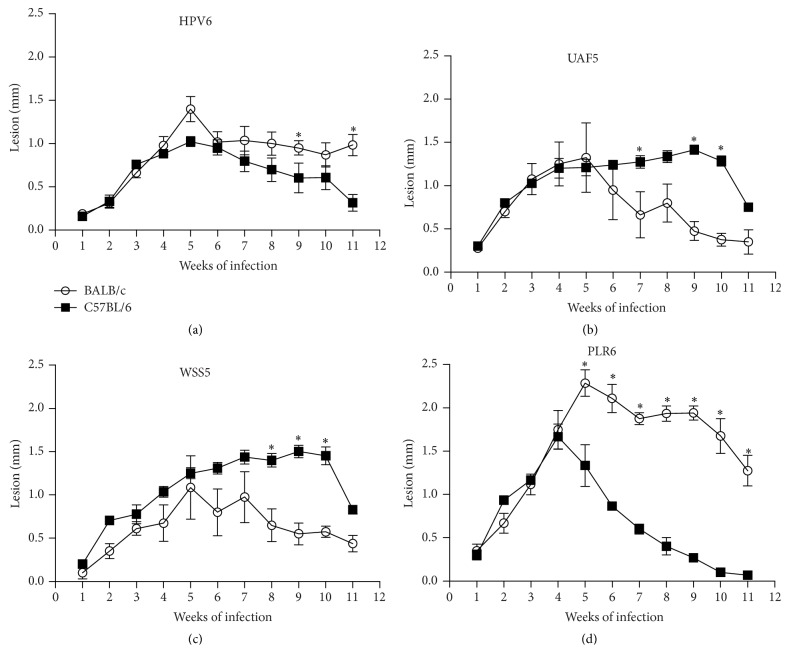
Time course of lesion development in C57Bl/6 WT (■) and BALB/c (○) mice infected with* Leishmania (V.) braziliensis* (HPV6, UAF5, and WSS5) or* Leishmania (V.) guyanensis* (PLR6). Mice were infected with promastigotes in stationary phase of growth (5 × 10^6^ parasites) into hind footpads. The lesion size was expressed in mm (infected minus control contralateral paw thickness). Data represent mean ± SEM of results from two or three independent experiments (4–14 animals): HPV6 (a), UAF5 (b), WSS5 (c), and PLR6 (d); ^*∗*^ *P* < 0.05.

In order to compare the outcome of infection caused by all isolates, stationary-growth-phase promastigotes (the 6th day of culture) were inoculated into C57BL/6 WT and BALB/c mouse footpads. Infection was successfully established for all* L. (V.) braziliensis* isolates in C57BL/6 WT and BALB/c mice and the lesions increased to a size of approximately 1.0–1.5 mm (Figures 3(a), 3(b), and 3(c)). The infection with the* L. (V.) guyanensis* PLR6 isolate caused a lesion more severe in BALB/c mice than in C57BL/6 (*P* < 0.05), which completely controlled the infection by 11 weeks (Figure 3(d)).

**Figure 4:**
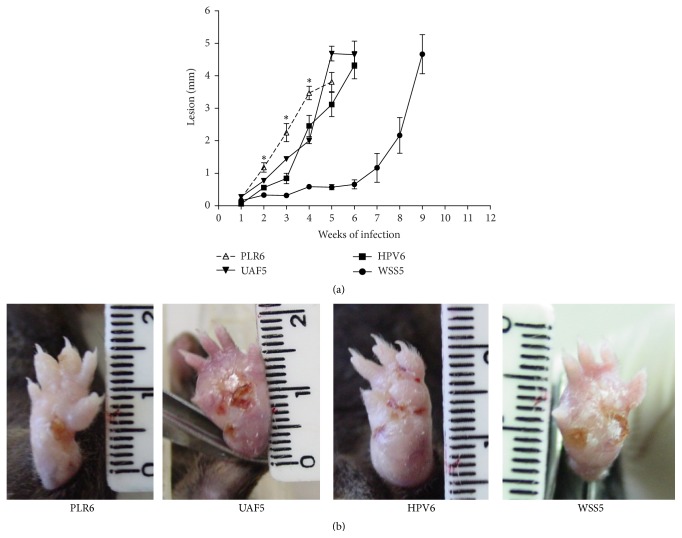
Time course of lesion development in IFN*γ*-deficient C57BL/6 mice infected with* L. (V.) braziliensis* (HPV6, UAF5, and WSS5) or* L. (V.) guyanensis* (PLR6). Lesion size was expressed in mm (infected minus control contralateral paw thickness). Data represent mean ± SEM (3–10 animals, (a)), ^*∗*^ *P* < 0.05. In (b), lesions caused by* L. (V.) braziliensis* (HPV6, UAF5, and WSS5) or* L. (V.) guyanensis* (PLR6).

In the absence of IFN*γ* (IFN*γ* KO C57BL/6), progressive lesions developed rapidly, except for the* L. (V.) braziliensis* WSS5 isolate (Figure 4(a)). Both* L. (V.) braziliensis* and* L. (V.) guyanensis* caused ulcerated lesions in IFN*γ* KO C57BL/6 (Figure 4(b)). In IFN*γ* KO C57BL/6 mice inoculated with* L. (V.) guyanensis* PLR6 isolate, lesions developed faster than in mice of the same strain inoculated with* L. (V.) braziliensis* (Figure 4(a)) and dissemination of parasites to the contralateral paw (increased thickness) was apparent on the 6th week after infection when the infected footpad began to ulcerate (data not shown).

### 3.5. Histopathological Analysis

**Figure 5:**
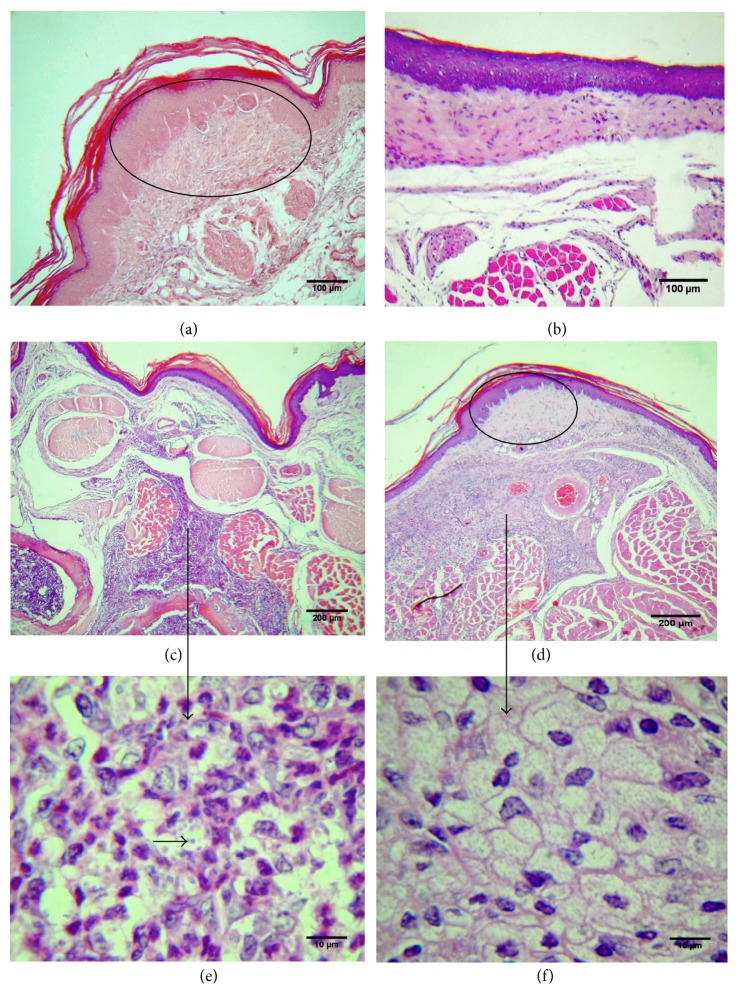
Photomicrographs of nonulcerated lesions obtained from wild-type C57BL/6 and BALB/c mice infected with* L. (V.) braziliensis* WSS5 or* L. (V.) guyanensis* PLR6. Mice were inoculated with 5 × 10^6^ parasites at stationary phase of growth, and 11 weeks after infection the histopathology of footpad lesions was evaluated after H&E staining. (a) C57BL/6 WT mouse infected with* L. (V.) braziliensis* WSS5, (b) C57BL/6 WT mouse infected with* L. (V.) guyanensis* PLR6, (c) BALB/c mouse infected with* L. (V.) braziliensis* WSS5 showing inflammatory infiltration in deep dermis, (d) BALB/c mouse infected with* L. (V.) guyanensis* PLR6 showing inflammatory infiltrate in deep dermis, (e) BALB/c mouse infected with* L. (V.) braziliensis* WSS5 (horizontal black arrow indicates the parasite; vertical black arrow indicates the infiltrate of deep dermis in (c) that contains macrophages and parasites), and (f) BALB/c mouse infected with* L. (V.) guyanensis* PLR6 (vertical black arrow indicates the mononuclear cell infiltrate in deep dermis in (d) that contains vacuolated macrophages without intact parasites). Areas of hypertrophic scar formation are indicated by the circles in (a) and (d).

**Figure 6:**
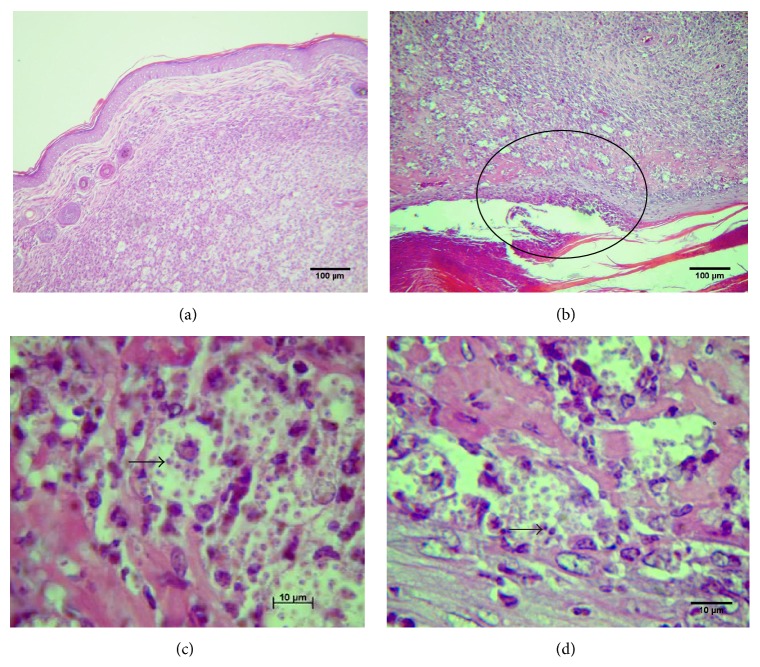
Photomicrographs of lesions caused by* L. (V.) braziliensis* WSS5 and* L. (V.) guyanensis* PLR6 in IFN*γ*-deficient C57BL/6 mice. Mice were inoculated with 5 × 10^6^ parasites in stationary phase of growth, and six weeks after infection the histopathology of footpad lesions was evaluated after H&E staining. (a) Nonulcerated lesion in mouse infected with* L. (V.) braziliensis* WSS5 isolate, (b) ulcerated lesion in mouse infected with* L. (V.) guyanensis* PLR6 (the circle shows part of the ulceration that contains mononuclear cells and parasites showed in (d)), (c) mononuclear cell infiltrate with presence of parasites of* L. (V.) braziliensis* WSS5 (black arrow), and (d) mononuclear cells infiltrate with presence of parasites of* L. (V.) guyanensis* PLR6 (black arrow).

Sections of the footpads obtained on the 11th week after infection with* L. (V.) braziliensis* WSS5 or* L. (V.) guyanensis* PLR6 were examined (Figures 5 and 6). On histological examination, the lesions in C57BL/6 footpads infected with either* Leishmania* isolate were characteristic of the late phase of tissue repair, with hypertrophic scar formation and marked fibrosis in the dermis with few mononuclear inflammatory cells; the epidermis was intact with hyperplasia of epidermal cells (Figures 5(a) and 5(b)). No parasites could be seen at higher magnification (1000x, data not shown).

In comparison, BALB/c mice infected with* L. (V.) braziliensis* also presented intact epidermis and superficial dermis but, in the deep dermis, a mononuclear inflammatory infiltrate rich in vacuolated macrophages was located close to and infiltrating the muscle bundles (Figure 5(c)); most macrophages were parasitized with* L. (V.) braziliensis* WSS5 (Figure 5(e)). On the other hand, in mice inoculated with* L. (V.) guyanensis* PLR6, hypertrophic scar and accentuated fibrosis were seen in the dermis and a mononuclear inflammatory infiltrate (Figure 5(d)) with vacuolated macrophages free of intact parasites was observed in the deep dermis (Figure 5(f)).

A marked difference in the histology was seen in IFN*γ* KO C57BL/6 on the 6th week after inoculation with the same isolates (Figure 6). These mice, inoculated with the* L. (V.) braziliensis* WSS5 isolate, did not have epidermal ulceration of the paw and from the plantar to dorsal side of the paw there was an infiltration of mononuclear cells with many parasite-laden macrophages (Figures 6(a) and 6(c)). In contrast, the footpads of mice inoculated with the* L. (V.) guyanensis* PLR6 isolate, as also the other* L. (V.) braziliensis* isolates, presented a visible ulceration in the plantar surface of the footpad; the inflammatory infiltrate was predominantly mononuclear with areas of necrosis and fibrin deposition near the base of the ulcer (Figures 6(b) and 6(d)); large numbers of parasite-laden macrophages were scattered in the whole dermis (Figure 6(d)).

## 4. Discussion

This report characterizes the* Leishmania (Viannia)* species isolated from skin biopsies of four patients assisted at the Tropical Diseases Hospital (Anuar Auad, Goiânia, Goiás), with a diagnosis of localized cutaneous leishmaniasis. In our previous studies, three of these isolates were identified as* L. (V.) braziliensis* (data not shown and [29]) and one remained unidentified. Here, characterization of the ribosomal ITS allowed identification of the latter isolate as* L. (V.) guyanensis*. It is important to stress here the difficulties in correctly identifying this isolate as* L. (V.) guyanensis*. To achieve this characterization, we have used three strategies: small subunit ribosomal RNA (SSU rDNA) was sequenced [24]; sets of primers for G6PD were used in PCR assays to discriminate* L. (V.) braziliensis* from other organisms of the* Viannia* subgenus [25]; and ITS was amplified and cloned and the nucleotide sequence of three independent positive clones was phylogenetically analyzed [28]. It is crucial to identify the* Leishmania* species in order to define which parasites are circulating in a geographic area, to establish the transmission cycles of ATL and to implement the best possible treatment. These points, especially the last one, together with our results, indicate the need of more suitable molecular techniques to define the* Leishmania* species in the diagnosis of ATL.

In this study, all ATL patients resided in Goiás, but the presumed geographic areas of patient infections for the three* L. (V.) braziliensis* isolates obtained were Goiás and Tocantins states. The patient infected with* L. (V.) guyanensis* reported having travelled to a forest zone in the boundary of the states Pará (Northern region) and Maranhão (Northeastern region) two months prior to the appearance of lesions. It is known that* L. (V.) braziliensis* is the parasite largely responsible for ATL in all five Brazilian regions, including Central Western, whereas* L. (V.) guyanensis* is prevalent only in the Northern region of Brazil, and there are no reports about autochthonous cases of ATL caused by this species in Goiás state or another Brazilian regions [7, 8]. Thus, our findings confirm the fact that there is a high probability of* L. (V.) guyanensis* being introduced in Goiás state due to the migratory behavior of patients infected with these parasites from the Northern to Central Western region. This possibility is reinforced by the fact that the Tropical Disease Hospital/Anuar Auad assists several patients from the states of the Northern region (around 25% of the assisted patients,* personal communication*), pointing out the need of parasite species identification in ATL patients.

The distribution of* Leishmania* spp. is dependent on vectors and reservoir hosts present in a geographic area. Thus, in Goiás, there are 47 different species of phlebotomine sand flies [14], with a predominance of* L. intermedia* and* L. whitmani* [8, 13, 14] which are vectors of* L. (V.) braziliensis* in Goiás, Tocantins, Pará, and Maranhão states. For* L. (V.) guyanensis*,* L. umbratilis* is the main vector in the Brazilian Northern region; it has not been reported in Goiás [8, 13, 14]. However,* L. umbratilis* is also* L. (V.) braziliensis* vector in Mato Grosso (Central Western region); and* L. whitmani*, which is associated with a great variety of vegetation, including Amazonian forest, Cerrado (savanna, predominant in Goiás), and Caatinga (Northeastern savanna), also transmits* L. (V.) guyanensis*. Moreover,* L. flaviscutellata*, present in Goiás, was found to transmit* L. (V.) guyanensis*. Besides, anteaters and opossums, considered as reservoir of* L. (V.) guyanensis*, are present in Goiás [1, 8, 14, 30–34].

Our findings prompted us to closely evaluate* L. (V.) guyanensis* which has so far been poorly investigated in Brazil. The clinical findings were similar between patients infected with* L. (V.) braziliensis* and with* L. (V.) guyanensis*. The patient infected with* L. (V.) guyanensis* presented three ulcerated lesions and received the treatment but did not return for a follow-up examination. The similarity between clinical manifestations in LCL caused by* L. (V.) braziliensis* and* L. (V.) guyanensis* has been reported, but the response to antimonial treatment can be different [15, 17]. The isolate* L. (V.) guyanensis* PLR6 has not been tested for antimonial susceptibility, but the other isolates* L. (V.) braziliensis* HPV6, UAF5, and WSS5 were uniformly susceptible* in vitro* to meglumine antimoniate and amphotericin B [22].

Corroborating the difficulties in identifying the* Leishmania* species relying on the clinical findings, our results did not show any difference in the monophasic-culture replication rates among the different isolates. In these cultures,* Leishmania* species were morphologically similar, and the* in vitro* growth profiles were similar to those previously described for* L. (V.) braziliensis* [34].

Our group has previously confirmed that the isolate* L. (V.) braziliensis* HPV6 is able to infect C57BL/6 mice and the J774 murine macrophage cell line [33]. In the present study, we confirmed the infection capacity of this isolate in C57BL/6 and BALB/c mice. All four isolates infected C57BL/6 and BALB/c mice. In contrast to C57BL/6 WT mice infected with* L. (V.) braziliensis* isolates, those infected with* L. (V.) guyanensis* PLR6 showed fast regression of the lesion, which almost disappeared after 11 weeks. BALB/c mice also showed nonulcerative skin swelling when infected with all isolates. However, in the deep dermis of* L. (V.) guyanensis* PLR6-infected mice, no parasites were detected inside macrophages whereas in* L. (V.) braziliensis* WSS5-infected footpads we observed a large number of parasites. The size of lesions caused by (*L. (V.) braziliensis*) HPV6, UAF5, and WSS5 was similar to those described by Pereira et al. [35] but larger than the size found in murine models of infection with this species [36–38]. de Moura et al. [39] reported that inoculation of* L. (V.) braziliensis* into the ear dermis of BALB/c mice leads to the development of an ulcerated lesion. The discrepancies with our results could be related to the site of inoculation or the virulence of the parasite strain. Considering the size and time course of the infection caused by* L. (V.) guyanensis* PLR6, our results were similar to those obtained by Sousa-Franco et al. [40], and like these authors we did not find parasites inside macrophages after 11 weeks of infection.

In this study, high susceptibility of IFN*γ* KO C57BL/6 mice to all four isolates confirms a close association between resistance and production of Th1 cytokines (IFN*γ*) during the course of* L. (Viannia)* spp. infection as has been described by de Souza-Neto et al. [41] in one* L. (V.) braziliensis* mouse model. The infection of IFN*γ* KO C57BL/6 showed macroscopical and microscopical differences between infected footpads of mice inoculated either with* L. (V.) braziliensis* or* L. (V.) guyanensis*. The development of the lesion caused by* L. (V.) guyanensis* PLR6 was faster than those caused by* L. (V.) braziliensis* isolates and PLR6 caused cutaneous metastatic lesions that could be observed in the contralateral footpad. The histopathology analysis showed parasites in the ulcerative area in lesions from the 5th week after infection. Secondary cutaneous metastatic lesions induced by* L. (V.) guyanensis* have also been reported in hamsters [42]. On the other hand, on the 5th week of infection, the whole extension of* L. (V.) braziliensis* WSS5-infected footpad consisted of an intense inflammatory infiltration full of parasites, without signs of ulceration; however, after nine weeks of infection, this parasite induced the same ulceration as the other* L. (V.) braziliensis* isolates. As C57BL/6 WT mice presented better control of the* L. (V.) guyanensis* infection and the development of the lesion caused by this species was faster than that caused by* L. (V.) braziliensis* in IFN*γ* KO C57BL/6 mice, it can be suggested that* L. (V.) guyanensis* can present higher susceptibility to leishmanicidal mechanisms induced by IFN*γ* than* L. (V.) braziliensis*. The highest susceptibility of IFN*γ* KO C57BL/6 to* L. (Viannia)* spp. led us to use this mouse strain for the process of parasite isolation from lesions of ATL patients [21, 43].

ATL has been considered as a social and economic problem of the poor population which has resulted largely from an intense migratory movement into rural areas and the forested hillsides that are close to the outskirts of the urban centers. However, due to the intense international travel and the large contingents of displaced and migratory populations, Tegumentary Leishmaniasis has to be considered as a diagnosis of nonhealing indolent ulcers also in nonendemic areas. Moreover, infection by* L. (V.) guyanensis* has been diagnosed in Europe in a soldier who denied travelling to endemic areas or having blood transfusions, raising some uncommon possibilities of contagion [44].

Nowadays, migration among Brazilian regions has largely increased, both inside forested areas and in urban areas. The finding of infection with* L. (V.) guyanensis* in a patient residing in the state of Goiás, where species other than* L. (V.) braziliensis* and* L. (L.) amazonensis* had not been previously described [7, 8, 45], improves the knowledge about ATL spreading pattern and reinforces the need for surveillance, control, and prevention of new ATL foci in Brazil. The difficulties to differentiate* L. (V.) guyanensis* from* L. (V.) braziliensis* at several levels, such as molecular, morphological, and clinical ones, draw attention to the possible underestimated prevalence of* L. (V.) guyanensis* in different Brazilian regions. Besides this contribution, our results also increased the knowledge on* L. (V.) guyanensis* infectivity in murine infection models, suggesting that IFN*γ* can be more relevant for controlling* L. (V.) guyanensis* than* L. (V.) braziliensis*.

## 5. Conclusions

We have isolated and characterized three clinical field isolates of* Leishmania* spp., from patients probably infected in Goiás, Tocantins, and Pará states, Brazil, as* L. (Viannia) braziliensis* and one as* L. (V.) guyanensis*. The latter species had not yet been described in Goiás. Infection of mouse strains BALB/c, C57BL/6 wild-type, and C57BL/6 lacking gamma-interferon (IFN*γ* KO C57BL/6) showed differences in lesion development among the* Leishmania* strains. In addition, better infection control of* L. (V.) guyanensis* than* L. (V.) braziliensis* was observed in mice in the presence of IFN*γ* but not in the absence of this cytokine. Molecular identification of* L. (V.) guyanensis* in a patient resident in Goiás stresses the importance of correct species identification and suggests that the presence of this species is possibly underestimated in several areas of Brazil.

## Figures and Tables

**Table 1:** ATL patients from whom the isolates were obtained^a^.

Patients	HPV	UAF	WSS	PLR
Sex^b^	M	M	M	M
Age^c^	46	29	22	19
Clinical form^d^	CL	CL	CL	CL
Number of lesions	2	1	1	3
Type of lesions	Ulcerated	Ulcerated	Ulcerated	Ulcerated
Time of lesion^e^	3 m	2 m	8 m	2 m
Lesion site	Upper limbs	Lower limb	Lower limb	Lower limbs
Satellite adenomegaly	No (lymphangitis)	No	No	No
Leishmanin skin test	No reaction	5 mm	NR^f^	5 mm
Histopathological analysis	Presence of amastigotes	Presence of amastigotes	NR	Presence of amastigotes
Treatment	Pentavalent antimonial	Pentavalent antimonial (two cycles)	No treatment	Pentavalent antimonial
Indirect immunofluorescence reaction	No reaction	No reaction 80 (after treatment)	NR	160
Clinical outcome	Clinical cure	Clinical cure	No treatment	NR
Presumed place of infection	Tocantins (TO)^g^	Tocantins (TO)	Goiás (GO)^h^	Pará^i^/Maranhão (MA)^j^ (PA)

^a^Patients were assisted at Anuar Auad Tropical Disease Hospital, Goiânia, Goiás (2005-2006).

^b^F = feminine, M = masculine, ^c^age in years, ^d^clinical form CL = cutaneous localized, ^e^time of lesion = in months, and ^f^NR = patient did not return. ^g^Tocantins (TO) is a state of the Northern region, ^h^Goiás (GO) is a state in Central Western region, ^i^Pará (PA) is a state of the Northern region, and ^j^Maranhão (MA) is a state of the Northeastern region (border with Pará).

## References

[1] Ashford R. W. (2000). The leishmaniases as emerging and reemerging zoonoses. *International Journal for Parasitology*.

[2] Reithinger R., Dujardin J.-C., Louzir H., Pirmez C., Alexander B., Brooker S. (2007). Cutaneous leishmaniasis. *Lancet Infectious Diseases*.

[3] Alvar J., Vélez I. D., Bern C. (2012). Leishmaniasis worldwide and global estimates of its incidence. *PLoS ONE*.

[4] Ministério da Saúde (2007). *Manual de Vigilância da Leishmaniose Tegumentar Americana*.

[5] SINAN/SVS/MS Coeficiente de detecção de casos de Leishmaniose Tegumentar Americana por 100.000 habitantes. Brasil, Grandes Regiões e Unidades Federadas. 1990 a 2010. http://portal.saude.gov.br/portal/arquivos/pdf/lta_deteccao_08_09_11.pdf.

[6] SINAN/SVS/MS (2013). *Casos de Leishmaniose Tegumentar Americana. Brasil, Grandes Regiões e Unidades Federadas. 1990 a 2013*.

[7] Grimaldi G., David J. R., McMahon-Pratt D. (1987). Identification and distribution of New World *Leishmania* species characterized by serodeme analysis using monoclonal antibodies. *The American Journal of Tropical Medicine and Hygiene*.

[8] Ministério da Saúde (2010). *Manual de Vigilância da Leishmaniose Tegumentar Americana*.

[9] Cupolillo E., Momen H., Grimaldi G. (1998). Genetic diversity in natural populations of new world *Leishmania*. *Memorias do Instituto Oswaldo Cruz*.

[10] Cupolillo E., Brahim L. R., Toaldo C. B. (2003). Genetic polymorphism and molecular epidemiology of *Leishmania (Viannia) braziliensis* from different hosts and geographic areas in Brazil. *Journal of Clinical Microbiology*.

[11] Azevedo A. C. R., Souza N. A., Meneses C. R. V. (2002). Ecology of sand flies (*Diptera: Psychodidae: Phlebotominae*) in the north of the State of Mato Grosso, Brazil. *Memorias do Instituto Oswaldo Cruz*.

[12] Missawa N. A., Maciel G. B. M. L., Rodrigues H. (2008). Geographical distribution of *Lutzomyia* (*Nyssomyia*) *whitmani* (Antunes & Coutinho, 1939) in the State of Mato Grosso. *Revista da Sociedade Brasileira de Medicina Tropical*.

[13] Martins F., Da Silva I. G., Bezerra W. A. (2002). Diversidade e frequencia da fauna flebotomínea (*Diptera*: *Psychodidae*) em áreas com transmissão de leishmaniose, no estado de Goiás. *Revista de Patologia Tropical*.

[14] de Almeida P. S., de Andrade A. J., Sciamarelli A. (2015). Geographic distribution of phlebotomine sandfly species (*Diptera: Psychodidae*) in Central-West Brazil. *Memórias do Instituto Oswaldo Cruz*.

[15] Romero G. A. S., Guerra M. V., Paes M. G., Macêdo V. O. (2001). Comparison of cutaneous leishmaniasis due to *Leishmania (Viannia) braziliensis* and *L. (V.) guyanensis* in Brazil: therapeutic response to meglumine antimoniate. *The American Journal of Tropical Medicine and Hygiene*.

[16] Couppié P., Clyti E., Sainte-Marie D., Dedet J. P., Carme B., Pradinaud R. (2004). Disseminated cutaneous leishmaniasis due to *Leishmania guyanensis*: case of a patient with 425 lesions. *American Journal of Tropical Medicine and Hygiene*.

[17] Arevalo J., Ramirez L., Adaui V. (2007). Influence of *Leishmania* (*Viannia*) species on the response to antimonial treatment in patients with American tegumentary leishmaniasis. *Journal of Infectious Diseases*.

[18] Guerra J. A. D. O., Prestes S. R., Silveira H. (2011). Mucosal Leishmaniasis caused by *Leishmania* (*Viannia*) *braziliensis* and *Leishmania* (*Viannia*) *guyanensis* in the Brazilian Amazon. *PLoS Neglected Tropical Diseases*.

[19] Schriefer A., Schriefer A. L. F., Góes-Neto A. (2004). Multiclonal *Leishmania braziliensis* population structure and its clinical implication in a region of endemicity for American tegumentary leishmaniasis. *Infection and Immunity*.

[20] Boité M. C., Mauricio I. L., Miles M. A., Cupolillo E. (2012). New insights on taxonomy, phylogeny and population genetics of *Leishmania (Viannia)* parasites based on multilocus sequence analysis. *PLoS Neglected Tropical Diseases*.

[21] de Oliveira M. A. P., Pires A. D. S., de Bastos R. P. (2010). *Leishmania spp*. parasite isolation through inoculation of patient biopsy macerates in interferon gamma knockout mice. *Revista do Instituto de Medicina Tropical de São Paulo*.

[22] Zauli-Nascimento R. C., Miguel D. C., Yokoyama-Yasunaka J. K. U. (2010). *In vitro* sensitivity of *Leishmania (Viannia) braziliensis* and *Leishmania (Leishmania) amazonensis* Brazilian isolates to meglumine antimoniate and amphotericin B. *Tropical Medicine and International Health*.

[23] Mouriz Savani E. S. M., Brandão Nunes V. L., Bianchi Galati E. A. (2005). Ocurrence of co-infection by *Leishmania (Leishmania)* chagasi and *Trypanosoma (Trypanozoon)* evansi in a dog in the state of Mato Grosso do Sul, Brazil. *Memorias do Instituto Oswaldo Cruz*.

[24] Uliana S. R. B., Nelson K., Beverley S. M., Camargo E. P., Floeter-Winter L. M. (1994). Discrimination amongst *Leishmania* by polymerase chain reaction and hybridization with small subunit ribosomal DNA derived oligonucleotides. *Journal of Eukaryotic Microbiology*.

[25] Castilho T. M., Shaw J. J., Floeter-Winter L. M. (2003). New PCR assay using glucose-6-phosphate dehydrogenase for identification of *Leishmania* species. *Journal of Clinical Microbiology*.

[26] Cupolillo E., Grimaldi G., Momen H., Beverley S. M. (1995). Intergenic region typing (IRT): a rapid molecular approach to the characterization and evolution of *Leishmania*. *Molecular and Biochemical Parasitology*.

[27] Schönian G., Nasereddin A., Dinse N. (2003). PCR diagnosis and characterization of Leishmania in local and imported clinical samples. *Diagnostic Microbiology and Infectious Disease*.

[28] Stamatakis A. (2014). RAxML version 8: a tool for phylogenetic analysis and post-analysis of large phylogenies. *Bioinformatics*.

[29] Depledge D. P., MacLean L. M., Hodgkinson M. R. (2010). *Leishmania*-specific surface antigens show sub-genus sequence variation and immune recognition. *PLoS Neglected Tropical Diseases*.

[30] da Costa S. M., Cechinel M., Bandeira V., Zannuncio J. C., Lainson R., Rangel E. F. (2007). *Lutzomyia* (*Nyssomyia*) *whitmani* s.l. (Antunes & Coutinho, 1939)(*Diptera: Psychodidae: Phlebotominae*): geographical distribution and the epidemiology of American cutaneous leishmaniasis in Brazil—mini-review. *Memorias do Instituto Oswaldo Cruz*.

[31] Shaw J. (2007). The leishmaniases—survival and expansion in a changing world. A mini-review. *Memorias do Instituto Oswaldo Cruz*.

[32] Fouque F., Gaborit P., Issaly J. (2007). Phlebotomine sand flies (*Diptera: Psychodidae*) associated with changing patterns in the transmission of the human cutaneous leishmaniasis in French Guiana. *Memórias do Instituto Oswaldo Cruz*.

[33] Leite P. M., Gomes R. S., Figueiredo A. B. (2012). Ecto-nucleotidase activities of promastigotes from *Leishmania (Viannia) braziliensis* relates to parasite infectivity and disease clinical outcome. *PLoS Neglected Tropical Diseases*.

[34] da Silva I. A., Morato C. I., Quixabeira V. B. L. (2015). *In vitro* metacyclogenesis of *Leishmania (Viannia) braziliensis* and *Leishmania (Leishmania) amazonensis* clinical field isolates, as evaluated by morphology, complement resistance, and infectivity to human macrophages. *BioMed Research International*.

[35] Pereira C. G., Silva A. L. N., de Castilhos P. (2009). Different isolates from *Leishmania braziliensis* complex induce distinct histopathological features in a murine model of infection. *Veterinary Parasitology*.

[36] Maioli T. U., Takane E., Arantes R. M. E., Fietto J. L. R., Afonso L. C. C. (2004). Immune response induced by New World *Leishmania* species in C57BL/6 mice. *Parasitology Research*.

[37] Indiani de Oliveira C., Teixeira M. J., Teixeira C. R. (2004). *Leishmania braziliensis* isolates differing at the genome level display distinctive features in BALB/c mice. *Microbes and Infection*.

[38] Rocha F. J. S., Schleicher U., Mattner J., Alber G., Bogdan C. (2007). Cytokines, signaling pathways, and effector molecules required for the control of *Leishmania (Viannia) braziliensis* in mice. *Infection and Immunity*.

[39] de Moura T. R., Novais F. O., Oliveira F. (2005). Toward a novel experimental model of infection to study American cutaneous leishmaniasis caused by *Leishmania braziliensis*. *Infection and Immunity*.

[40] Sousa-Franco J., Araújo-Mendes É., Silva-Jardim I. (2006). Infection-induced respiratory burst in BALB/c macrophages kills *Leishmania guyanensis* amastigotes through apoptosis: possible involvement in resistance to cutaneous leishmaniasis. *Microbes and Infection*.

[41] de Souza-Neto S. M., Carneiro C. M., Vieira L. Q., Afonso L. C. C. (2004). *Leishmania braziliensis*: partial control of experimental infection by interleukin-12 p40 deficient mice. *Memorias do Instituto Oswaldo Cruz*.

[42] Martinez J. E., Travi B. L., Valencia A. Z., Saravia N. G. (1991). Metastatic capability of *Leishmani* (*Viannia*) *panamensis* and *Leishmania* (*Viannia*) *guyanensis* in golden hamsters. *Journal of Parasitology*.

[43] Dorta M. L., Oliveira M. A. P., Fleuri A. K. A. (2012). Improvements in obtaining New World *Leishmania* sp from mucosal lesions: notes on isolating and stocking parasites. *Experimental Parasitology*.

[44] Poeppl W., Burgmann H., Auer H., Mooseder G., Walochnik J. (2012). *Leishmania (Viannia) guyanensis* infection, Austria. *Emerging Infectious Diseases*.

[45] Dorta M. L., Gosch C. S., Dorta R. O., Pereira L. I. A., Pereira A. J. S., Ribeiro-Dias F. (2003). American cutaneous leishmaniasis in a rural area of Goiás state, Brazil. *Revista do Instituto de Medicina Tropical de São Paulo*.

